# Dampened circadian amplitude of EEG power in women after menopause

**DOI:** 10.1111/jsr.14219

**Published:** 2024-04-26

**Authors:** Rafael Pérez‐Medina‐Carballo, Anastasi Kosmadopoulos, Christophe Moderie, Philippe Boudreau, Manon Robert, Diane B. Boivin

**Affiliations:** ^1^ Integrated Program in Neuroscience McGill University Montreal Quebec Canada; ^2^ Centre for Study and Treatment of Circadian Rhythms, Douglas Mental Health University Institute, Department of Psychiatry McGill University Montreal Quebec Canada; ^3^ Appleton Institute for Behavioural Sciences Central Queensland University Adelaide South Australia Australia; ^4^ Centre de Recherche du Centre Hospitalier de l'Université de Montréal, Université de Montréal Montreal Quebec Canada

**Keywords:** aging, circadian rhythms, electroencephalography power, menopause, sleep

## Abstract

Postmenopausal women are at high risk of developing sleep–wake disturbances. We previously reported dampened circadian rhythms of melatonin, alertness and sleep in postmenopausal compared with young women. The present study aims to further explore electroencephalography power spectral changes in the sleep of postmenopausal women. Eight healthy postmenopausal women were compared with 12 healthy, naturally ovulating, young women in their mid‐follicular phase. Participants followed a regular 8‐hr sleep schedule for ≥ 2 weeks prior to laboratory entry. The laboratory visit included an 8‐hr baseline sleep period followed by an ultradian sleep–wake cycle procedure, consisting of alternating 1‐hr wake periods and nap opportunities. Electroencephalography power spectral analysis was performed on non‐rapid eye movement sleep obtained over a 48‐hr period. The baseline nocturnal sleep of postmenopausal women comprised lower power within delta and sigma, and higher power within alpha bands compared with that of younger women. During nighttime naps of the ultradian sleep–wake cycle procedure, lower power within delta and sigma, and higher power within beta bands were observed in postmenopausal women. During the ultradian sleep–wake cycle procedure, postmenopausal women presented lower power of delta, theta and sigma (14‐15 Hz), undetectable rhythms of delta and theta, and a dampened or undetectable rhythm of sigma (12‐15 Hz) power compared with younger women. Our results support the hypothesis of a dampened circadian variation of sleep microstructure in healthy‐sleeping postmenopausal women. Circadian changes with aging are potential mechanisms for increased susceptibility to develop sleep disturbances; however, further research is needed to clarify their clinical implications and contribution to insomnia.

## 1 INTRODUCTION

Women are at a greater risk than men of developing sleep disturbances, both during and after the menopausal transition (Zolfaghari et al., 2020). Sleep disturbances increase after menopause and various factors contribute to this increase, including a higher prevalence of vasomotor symptoms, sleep disorders such as sleep‐disordered breathing and periodic leg movements (PLMS), as well as several chronic and mental health conditions (Arnardottir et al., 2016; Pennestri et al., 2006; Xu & Lang, 2014). We previously investigated changes in the circadian variation of sleep and alertness as contributing factors to the sleep disturbances of postmenopausal women (Perez‐Medina‐Carballo et al., 2023). We found that postmenopausal women had more disrupted sleep, slept more during daytime, and were more alert at night compared with younger women. We also found a dampened circadian amplitude of sleep‐onset latency (SOL), total sleep time (TST), stage N3 sleep, alertness and melatonin levels in postmenopausal women. These findings are consistent with a weakened circadian signal regulating sleep, alertness and melatonin secretion (Dijk & Duffy, 2020; Perez‐Medina‐Carballo et al., 2023).

In addition to the visually‐scored sleep stages, studying the microstructure of sleep measured by electroencephalography (EEG) power provides a more refined analysis of sleep (Feige et al., 2013). Existing research about sleep EEG changes after menopause is scarce (Campbell et al., 2011; Kalleinen, Polo‐Kantola, et al., 2008; Matthews et al., 2021), which is a knowledge gap given their potential contribution to the sleep disturbances commonly developed during this period of life. One longitudinal study showed that women who transitioned to a postmenopausal stage had increased beta power during non‐rapid eye movement (NREM) sleep compared with women who remained pre‐ or early perimenopausal (Matthews et al., 2021). In that study, no postmenopausal changes were observed in delta power, sleep duration or wake after sleep onset (WASO). Campbell et al. (2011), in a cross‐sectional study, found higher beta power in NREM and rapid eye movement (REM) sleep in late perimenopausal and postmenopausal women compared with premenopausal and early perimenopausal women, but no differences in delta power as a result of menopausal status. In another cross‐sectional study, Kalleinen et al. (2008) found that pre‐ and postmenopausal women had less slow‐wave activity (SWA; or delta power) than younger women (beta power not reported). While two studies found higher beta power, results for delta power were inconsistent, and other EEG frequency bands such as theta, alpha or sigma power were not measured. A cross‐sectional study including women and men (aged between 18 and 64 years) found that women had greater delta, theta and sigma power than men at all ages, with a similar age decline in both sexes. In women, an age‐related decrease in delta, theta and sigma with no differences in alpha or beta power have been described, but menopausal status was not considered (Schwarz et al., 2017; Svetnik et al., 2017).

Although increased beta and decreased delta power have been described at menopause, a definitive consensus remains elusive as menopausal status has not been consistently considered in aging studies. The aim of the present study is to better understand the changes occurring after menopause in EEG power frequency bands and their circadian variation as these might predispose women to experience sleep disturbances.

## 2 METHODS

### 2.1 Participants

Participant demographics, recruitment and screening methods, study design, and laboratory conditions have previously been described in Perez‐Medina‐Carballo et al. (2023). Briefly, participants included eight healthy postmenopausal women (mean age ± SD: 54.80 ± 3.37 years, range 50–61 years, at least 2 years since their last menstrual period) and 12 healthy young naturally‐ovulating women from a prior study, studied in their mid‐follicular phase (age: 25.83 ± 3.35 years, range 20–30 years; days 5–9 after menses; Shechter et al., 2010). All women were good sleepers based on a clinical evaluation by a sleep disorder physician (DBB) and verified by a polysomnographic (PSG) screening night at the laboratory. During screening procedures, postmenopausal women also reported good sleep quality based on the Insomnia Severity Index (mean ± SD: 3.75 ± 2.96) and the Pittsburgh Sleep Quality Index (3.25 ± 2.60). As reported in Pérez‐Medina‐Carballo et al. (2023), we previously showed that this group of postmenopausal women had comparable self‐reported sleep quality to young women throughout the ultradian sleep–wake cycle (USW) procedure (described below). All participants were physically and psychologically healthy. Participants were not using medications or hormonal therapy, apart from one postmenopausal woman who was using estradiol transdermal patches and micronized progesterone pills daily. Both groups were screened for sleep apnea (apnea–hypopnea index < 15 per hr of sleep in postmenopausal women; < 5 in young women; Table A1) and PLMS (index < 15 per hr of sleep). All procedures were approved by the Research Ethics Board of the Montreal West Island Integrated University Health and Social Services Centre (no. 2018‐175), and each participant provided informed consent.

### 2.2 Design

Prior to laboratory entry, participants maintained a regular 8‐hr sleep schedule for ≥ 2 weeks, confirmed by wrist actigraphy. This preparatory phase was planned to reduce variability in circadian entrainment between participants. Sleep schedules were discussed with each participant during the screening phase and were selected based on their regular sleep habits. Participants were admitted to the laboratory for an 8‐hr baseline sleep period at their habitual sleeping time. This was immediately followed by a USW procedure lasting 48 hr (postmenopausal women) or 72 hr (young women). The USW procedure consisted of alternating 1‐hr wake periods and 1‐hr nap opportunities in constant conditions. This procedure ended with an ad libitum nap.

During the laboratory visit, participants remained in a time‐isolation room without windows or time cues. Participants were exposed to dim light (< 10 lux) during wake periods of the USW procedure and complete darkness (~0 lux) during sleep opportunities. Food intake was divided into isocaloric snacks throughout wake periods of the USW procedure. Ambient temperature was maintained at 22.0 ± 2.0°C. Young women maintained a semi‐recumbent position throughout the USW procedure and were required to use a bed pan. Due to the risk of thrombophlebitis, postmenopausal women stayed in a semi‐recumbent position throughout the USW procedure, but were permitted to use the toilet in an ensuite bathroom every wake period and walk around the bedroom for 10 min every other wake period. Additionally, an anticoagulant was administered three times in the postmenopausal woman taking hormonal replacement therapy (2 ml tinzaparin sodium – Innohep, once per morning).

### 2.3 Measures

Sleep was PSG recorded (Harmonie, Stellate Systems, Canada) throughout baseline sleep and naps of the USW procedure. PSG recordings included EEG (F3/A2, F4/A1, C3/A2, C4/A1, O1/A2, O2/A1 for postmenopausal women; C3/A2, C4/A1, O1/A2, O2/A1 for young women), electrooculogram, electromyogram and electrocardiogram. PSG data were sampled with a frequency of 250 Hz in 11 young women and 512 Hz in postmenopausal women and one young woman. In order to compare data that were collected with 250 and 512 Hz, a resampling method based on linear interpolation was used (see details in Statistical Analyses). Sleep was visually scored in 30‐s epochs based on the American Academy of Sleep Medicine scoring guidelines (Berry et al., 2020) using central and occipital leads. For the baseline sleep period, sleep parameters included SOL, TST, sleep efficiency (SE), NREM sleep and WASO. These sleep parameters were calculated based on time in bed, and were previously reported (Perez‐Medina‐Carballo et al., 2023).

Automatic artifact removal was performed using HarmAct, an *ad‐hoc* software developed by “Sacré‐Cœur‐de‐Montréal” Hospital (Montreal, QC, Canada; Provencher et al., 2020), and was confirmed by visual inspection of each recording. Spectral band power was calculated using Fast Fourier Transform with a 4‐s Hamming window with 50% overlap between two adjacent windows. EEG spectral analysis was performed on NREM sleep stages of the central derivation C3. EEG frequency bands were categorized as follows: delta or SWA (0.5–4.5 Hz), theta (4.75–7.5 Hz), alpha (8–12 Hz), sigma or spindle frequency activity (SFA; 12–16 Hz), and beta (16–24.5 Hz). Some of the sigma power bands correspond to a range of low SFA (LSFA; 12.25–13.75 Hz) and others correspond to a range of high SFA (HSFA; 14–15.5 Hz) as defined by Munch and colleagues (2010). As spindle frequencies were suggested to differ between young and older women (Purcell et al., 2017), additional analyses were conducted with sigma power (12–16 Hz) divided into 1‐Hz bands. This allowed us to clarify the circadian variation in sigma power.

### 2.4 Statistical analyses

All statistical analyses were performed in R version 4.2.1. Absolute spectral power was calculated for the baseline sleep period and USW naps. Only the 24 naps from the first 48 hr of the USW were compared between groups. To analyse data collected at different sampling rates (i.e., 250 or 512 Hz), data from young women (250 Hz) were interpolated using a linear method to estimate their value at 512 Hz using the “prospectr” package (Stevens & Ramirez‐Lopez, 2022). After resampling, young women's data were expressed at a sampling frequency of 512 Hz, comparable to those of postmenopausal women. Using the package “lme4” (Bates et al., 2015), linear mixed‐effects models were used to compare EEG spectral power within and between groups. EEG power spectral analysis was performed using bins of 0.25 Hz as well as standard frequency bands (delta, theta, alpha, sigma, beta).

The EEG power of the baseline sleep was analysed as a full night and also divided into thirds based on the duration of the sleep period (from sleep onset to final awakening) to observe time‐dependent changes (Svetnik et al., 2017). The baseline sleep period of one young woman was excluded due to technical problems. USW recordings were categorized into daytime (nap episodes 1–8 and 13–20) and nighttime (nap episodes 9–12 and 21–24) naps, corresponding to each participant's projected habitual sleep schedule.

Because data were not normally distributed, EEG power for the various frequency bands were log‐transformed for statistical analyses and used as dependent variables in separate linear mixed‐effect models: baseline sleep, daytime naps and nighttime naps. EEG power was binned in 0.25 Hz increments. The 0.25‐Hz intervals are labelled based on the lower end of the range. For example, the frequency bin named 0.75 Hz includes the power between ≥ 0.75 and < 1 Hz. Participant “group” (postmenopausal women versus young women) and “frequency bin” (in 0.25 Hz) were used as fixed effects. Participant numbers were included as a random effect. A forward stepwise method (Olive, 2017) was used to build the model and likelihood‐ratio tests were used to determine significance of the fixed effect when added to the model, as previously used in Perez‐Medina‐Carballo et al. (2023). Tukey's *post‐hoc* tests were performed when group × frequency interactions were significant. Additionally, to investigate the relationship between daytime and nighttime power spectra of the USW, nighttime data were transformed as percentage of daytime power as follows: [(nighttime EEG power/daytime EEG power) × 100].

Circadian parameters (mesor, amplitude and acrophase) were calculated for each frequency band (delta, theta, alpha, sigma, beta) to analyse their circadian variation throughout the naps of the USW. Circadian parameters were calculated based on the time elapsed into the USW procedure, which was aligned to each participant's habitual sleep period. The mesor was defined as the average value of the fitted or unfitted rhythm. Because we added a time elapsed into the USW linear effect into the model (explained below), the average values changed with time into the USW. Therefore, the mesor corresponded to the intercept value in the middle of the USW procedure. The acrophase was defined as the peak time of the fitted rhythm. The amplitude was defined as half the difference between the peak and trough levels. To analyse the circadian variation of brain activity in each EEG frequency band, data were Z‐scored using the mean and standard deviation of the baseline sleep period and then collapsed into 2‐hr bins. Cosinor analysis was then performed with a linear mixed‐effects model using a modified version of the package “cosinor” (Sachs, 2014) to include participant's number as random effects. “Group”, “frequency bands” (in Hz) and “time elapsed into the USW procedure” were used as fixed effects. A forward stepwise method (Olive, 2017) was utilized to build the model, and likelihood‐ratio tests were used to determine significance of the fixed effects added to the model. Tukey's *post‐hoc* tests were performed when group × frequency band interactions were significant.

Because we included one postmenopausal woman taking hormone replacement therapy, we evaluated her EEG power spectral results for outliers. Random effects estimates were extracted for each participant using the package “merTools” (Knowles et al., 2020). The random effects estimates of this postmenopausal woman remained within 2 SD for all parameters, and therefore her data were included in the analyses.

## 3 RESULTS

### 3.1 Baseline sleep period

Table 1 summarizes the parameters of the baseline sleep period. As reported previously, no significant group differences were observed in SOL, TST, SE, NREM sleep and WASO (Perez‐Medina‐Carballo et al., 2023). Postmenopausal women had earlier bedtimes (23:07 hours ± 00:11 versus 00:13 hours ± 12; *p* = 0.005) and rise‐times (07:07 hours ± 00:11 versus 08:13 hours ± 12; *p* = 0.005) than young women (Perez‐Medina‐Carballo et al., 2023).

**TABLE 1:** Baseline sleep parameters of postmenopausal and young women studied in their mid‐follicular phase.

	Young women (mean ± SEM)	Postmenopausal women (mean ± SEM)	*p*‐Value
SOL (min)	13.22 ± 1.66	13.94 ± 3.11	0.84
TST (min)	435.11 ± 4.01	424.00 ± 8.19	0.25
SE (%)	90.81 ± 0.93	88.82 ± 1.54	0.20
NREM (min)	336.27 ± 4.71	335.19 ± 8.93	0.92
WASO (min)	27.28 ± 3.79	37.56 ± 6.20	0.18

*Note*: All values are expressed as mean ± SEM. *p*‐Values were based on two‐tailed *t*‐test or Mann–Whitney *U*‐test when appropriate.

Abbreviations: NREM, non‐rapid eye movement sleep; SE, sleep efficiency; SOL, sleep‐onset latency; TST, total sleep time; WASO, wake after sleep onset.

**FIGURE 1:**
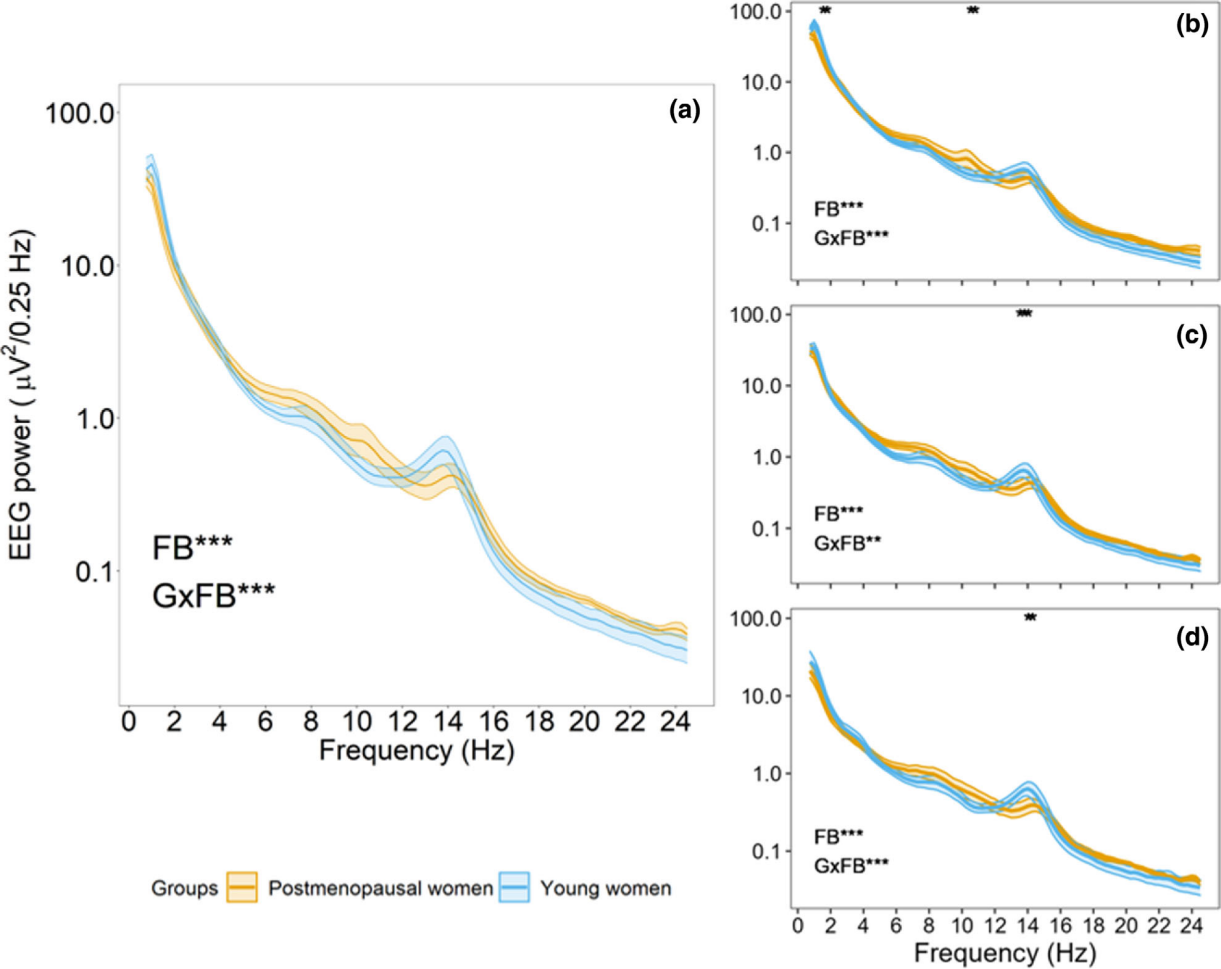
Electroencephalographic (EEG) power of the baseline nocturnal sleep period for the full night (a) and divided into thirds of the sleep period (b: first, c: second, d: third). FB, significant main effect of frequency bin; GxFB, significant group by frequency bin interaction. The asterisks (*) along the top *X*‐axis indicate significant group differences by frequency bin (*p* < 0.05). ***p < 0.001; **p < 0.01. Lines represent mean EEG power and shaded areas represent SEM.

Baseline EEG power density during NREM sleep is presented in Figure 1(a), and results of the linear mixed‐effects model are summarized in Table A2. Across the entire nighttime baseline sleep, a significant main effect of frequency bin (0.25 Hz bin) was observed (*p* < 0.001), but no main group effect (*p* = 0.54). The group by frequency bin interaction was significant (*p* < 0.001), but Tukey's *post‐hoc* tests revealed no significant differences between groups (*p* ≥ 0.060).

The baseline sleep period was further divided into thirds, and results are presented in Figure 1(b–d) and Table A2. In each third of the sleep period, the main effect of frequency bin (0.25 Hz bin; *p* < 0.001) and the group by frequency bin interaction were significant (*p* < 0.001), while the main effect of group was not (*p* ≥ 0.42). Tukey's *post‐hoc* tests revealed that, during the first third of the sleep period (Figure 1b), postmenopausal women showed significantly lower power for frequencies within the delta range (1–1.5 Hz; *p* ≤ 0.049) and higher power within the alpha range (10–10.5 Hz; *p* ≤ 0.040) than young women. During the second and last thirds of the sleep period (Figure 1c,d), postmenopausal women showed significantly lower power within the sigma range (13–14 Hz; *p* ≤ 0.049) than young women.

The EEG power values of standard frequency bands during the full baseline sleep period and divided by thirds are presented in Table A3. When baseline sleep was binned in standard frequency bands, only sigma power in the range of 13–14 Hz was significantly lower in postmenopausal compared with young women.

### 3.2 USW procedure

**FIGURE 2:**
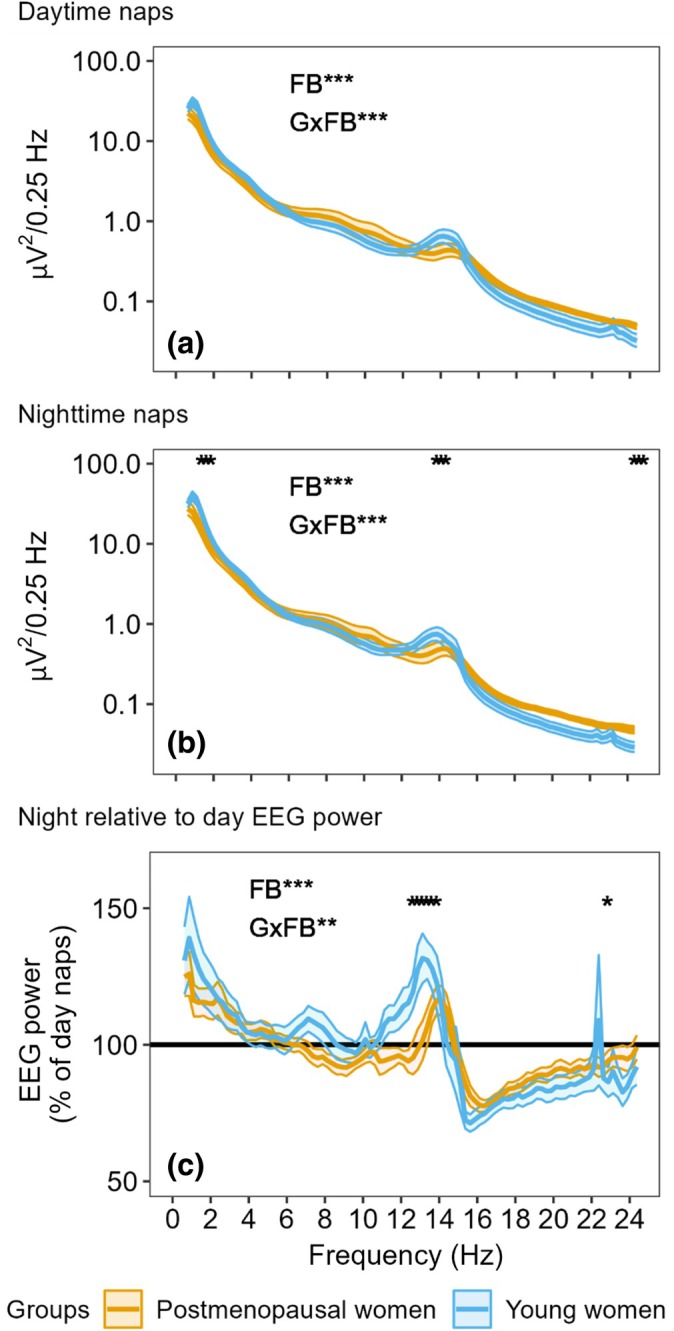
Electroencephalographic (EEG) power during daytime and nighttime naps of the ultradian sleep–wake cycle (USW) procedure. (a) EEG power during nighttime naps. (b) EEG power during daytime naps. (c) EEG power of nighttime naps relative to daytime naps. FB, significant main effect of frequency bin; GxFB, significant group by frequency bin interaction. The asterisks (*) along the top *X*‐axis indicate significant group differences by frequency bins (*p* < 0.05). ***p < 0.001, **p < 0.01. Lines represent mean EEG power, whereas shaded areas represent SEM.

Results of EEG power density during NREM sleep for the USW procedure are presented in Figure 2. During daytime naps (Figure 2a), a main effect of frequency bin (*p* < 0.001) and a group by frequency bin interaction (*p* < 0.001) were observed, but no main effect of group (*p* = 0.62). Tukey's *post‐hoc* tests did not reveal significant group differences during the daytime naps (*p* ≥ 0.062). During nighttime naps (Figure 2b), a main effect of frequency bin (*p* < 0.001) and a group by frequency bin interaction (*p* < 0.001) were observed, but no main effect of group (*p* = 0.58). For nighttime naps, Tukey's *post‐hoc* tests revealed a significantly reduced power for frequencies within the delta (0.75–1.5 Hz; *p* ≤ 0.047) and sigma (13.25–14 Hz; *p* ≤ 0.048) ranges, as well as increased power for frequencies within the beta range (23.75–24.5 Hz; *p* ≤ 0.042) in postmenopausal compared with young women.

The EEG power of nighttime relative to daytime naps was calculated to better depict the diurnal changes of spectral power by frequency band (Figure 2c). A main effect of frequency bin (*p* < 0.001) and a group by frequency bin interaction (*p* = 0.004) were observed, but no main effect of group. *Post‐hoc* tests revealed significantly lower power within the sigma range (12.00–13.5 Hz; *p* ≤ 0.030) and higher power within the beta range (22.25–22.5 Hz; *p* = 0.049) in postmenopausal compared with young women, in the nighttime relative to daytime. The nocturnal peak of LSFA occurred at higher frequencies in postmenopausal (~14 Hz) compared with young women (~13 Hz; Figure 2).

### 3.3 Circadian variation of frequency bands during the USW procedure

**FIGURE 3:**
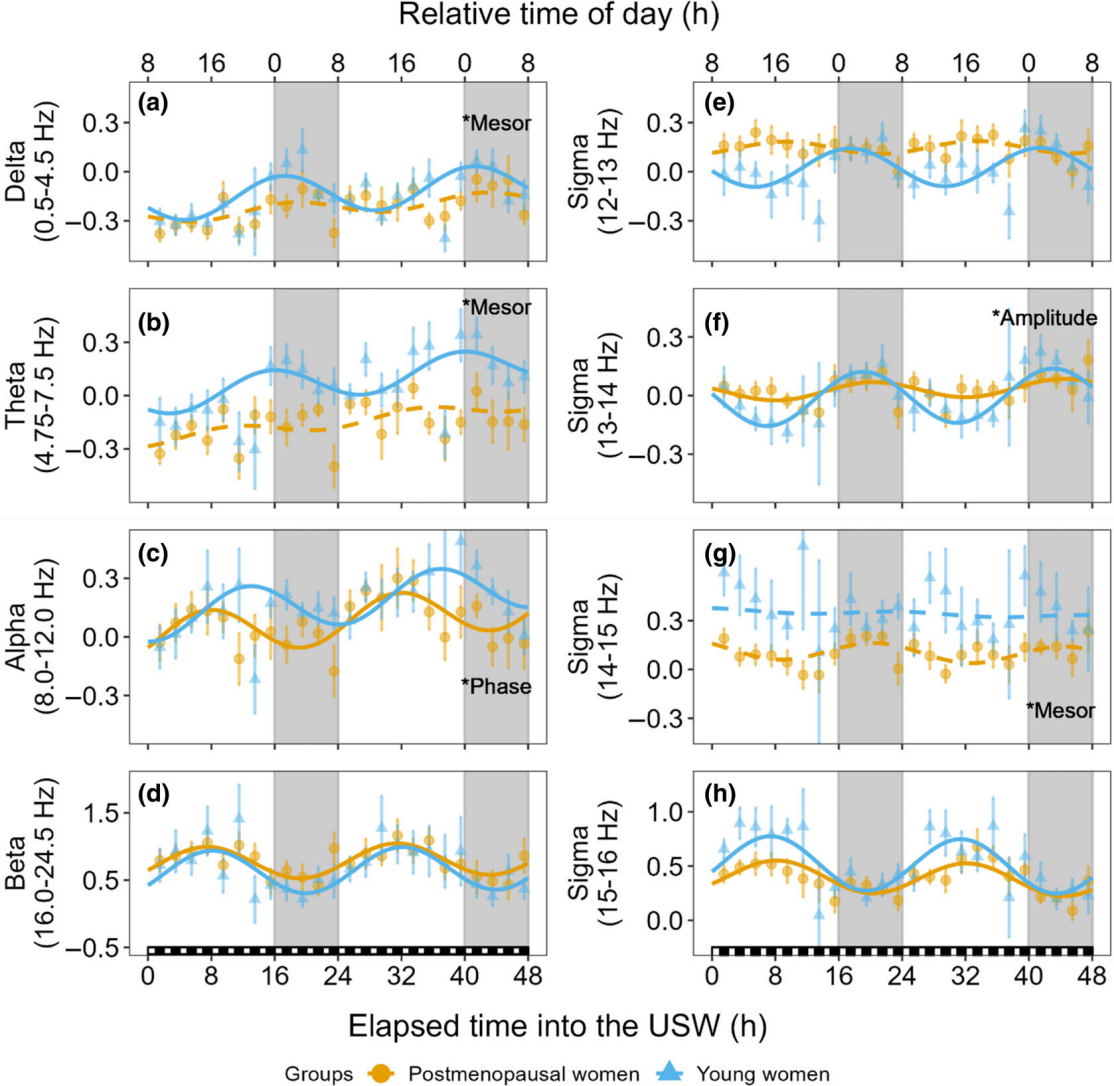
Circadian variation of electroencephalographic (EEG) spectral power per frequency band throughout the ultradian sleep–wake cycle (USW) procedure. Data were aligned on the time elapsed into the USW procedure (USW, bottom *X*‐axis). Solid lines represent significant cosinor regressions, whereas dashed lines depict non‐significant regressions. Black (~0 lux) and white (~10 lux) small squares above the bottom *X*‐axis represent the nap and wake periods across the first 48 hr of the USW procedure. The top *X*‐axis depicts the corresponding time of day for a participant with a hypothetical bedtime of 00:00 hours to 08:00 hours. The *Y*‐axis illustrates *Z*‐scores of frequency bands power. Large grey rectangles depict the projected time of the habitual nocturnal sleep period, which corresponds to the time between habitual bedtime and rise‐time. *Mesor = group differences in mesor, *Amplitude = group differences in amplitude, *Phase = group differences in phase. Values are presented as mean ± SEM.

The circadian variations of delta, theta, alpha, sigma and beta power across the USW procedure are presented in Figure 3. Results of the linear mixed‐effects model and cosinor regressions of delta, theta, alpha, sigma and beta power are reported in Tables 2 and 3, respectively. In both groups, a significant circadian variation was observed in alpha, sigma (at 13–14 and 15–16 Hz bins) and beta power. Young women additionally presented a significant circadian variation of delta, theta and sigma power (12–13 Hz bin). A lower mesor in delta, theta and sigma (14–15 Hz bin) power was observed in postmenopausal compared with young women. A lower circadian amplitude in sigma (13–14 Hz bin) power was observed in postmenopausal women, whereas the circadian amplitude of alpha and beta power was comparable between groups. Relative to rise‐time, postmenopausal women presented an earlier peak of the alpha power rhythm compared with young women. No other phase difference was observed in other frequency bands. The circadian parameters of frequency bands power are reported in Table 3. Finally, an effect of time elapsed into the USW was observed for delta, theta and alpha power, with increasing values across the USW procedure (*p* ≤ 0.016) and without group differences (*p* ≥ 0.19).

**TABLE 2:** Results of linear mixed‐effects model of EEG power frequency bands during the USW procedure.

Parameter	Group (*p*‐value)	Time into USW (*p*‐value)	Time into USW × group (*p*‐value)	Circadian variation (*p*‐value)	Circadian × group interaction (*p*‐value)
Delta power (0.5–4.5 Hz)	**0.045**	**0.002**	0.31	**0.011**	0.23
Theta power (4.75–7.5 Hz)	**0.008**	**< 0.001**	0.22	**0.017**	0.21
Alpha power (8–12 Hz)	0.30	**< 0.001**	0.92	**< 0.001**	**0.004**
Beta power (16–24.5 Hz)	0.51	0.56	0.88	**< 0.001**	0.43
Sigma power					
12–13 Hz	0.16	0.60	0.67	**0.023**	**< 0.001**
13–14 Hz	0.60	0.10	0.61	**< 0.001**	**0.003**
14–15 Hz	**0.051**	0.85	0.70	0.63	0.79
15–16 Hz	0.26	0.18	0.19	**< 0.001**	0.25

*Note*: Bold values denote statistical significance.

Abbreviation: USW, ultradian sleep–wake cycle.

[Correction added on 13 May 2024, after first online publication: The complete version of Table 2 has been added and the sequence of Table 2 and 3 has been corrected.]

**TABLE 3:** Circadian parameters of EEG power frequency bands in postmenopausal women (PMW) and young women studied at mid‐follicular phase (YW) based on time elapsed into the USW procedure.

Parameter	Mesor (z‐score)					Amplitude (*Z*‐score)							Acrophase (elapsed time into the USW in hr)				
	PMW		YW		*p*‐Value	PMW			YW			*p*‐Value	PMW		YW		*p*‐value
	Mean	SEM	Mean	SEM		Mean	SEM	95% CI	Mean	SEM	95% CI		Mean	SEM	Mean	SEM	
Delta power	−0.216	0.032	−0.129	0.029	**0. 045**	0.044	0.039	−0.033, 0.121	0.120	0.034	**0.052, 0.187**	0.14	–	–	16.976	0.817	‐
Theta power	−0.149	0.063	0.085	0.054	**0.008**	0.035	0.028	−0.020, 0.090	0.094	0.033	**0.029, 0.159**	0.18	–	–	15.387	1.245	‐
Alpha power	0.080	0.070	0.182	0.060	0.30	0.118	0.033	**0.053, 0.183**	0.119	0.025	**0.070, 0.169**	0.97	7.562	0.868	12.545	0.999	**<0.001**
Beta power	0.784	0.182	0.644	0.156	0.51	0.247	0.064	**0.121, 0.372**	0.329	0.057	**0.216, 0.441**	0.33	7.337	0.887	8.040	0.643	0.52
Sigma power (Hz)																	
12–13 Hz	0.147	0.068	0.026	0.058	0.16	0.039	0.024	−0.008, 0.085	0.116	0.019	**0.079, 0.154**	0.012	–	–	17.420	0.798	‐
13–14 Hz	0.029	0.052	−0.010	0.045	0.60	0.043	0.021	**0.002, 0.083**	0.135	0.018	**0.099, 0.171**	**< 0.001**	20.246	2.016	19.016	0.627	0.56
14–15 Hz	0.103	0.094	0.343	0.081	**0.051**	–	–	–	–	–	–	–	–	–	–	–	‐
15–16 Hz	0.389	0.078	0.512	0.067	0.26	0.144	0.045	**0.055, 0.233**	0.244	0.043	**0.159, 0.329**	0.11	8.232	1.038	7.452	0.551	0.51

*Note*: Circadian parameters were *Z*‐scored based on the mean and SD of the baseline sleep period. Positive mean values represent increased values in the USW relative to baseline sleep Negative mean values represent decreased values in the USW relative to baseline sleep. Amplitude and phase were calculated only when the circadian variation was significant. *p*‐Values for mesor are equivalent to the group effect in Table 2. *p*‐Values for amplitude and acrophase comparisons were based on two‐tailed *t*‐test. Bold values denote statistical significance.

Abbreviations: CI, confidence interval; PMW, postmenopausal women; USW, ultradian sleep–wake cycle; YW, young women.

The circadian variation of LSFA (12.25–13.75 Hz) and HSFA (14–15.5 Hz) across the USW procedure is presented in Figure A1. In LSFA, a significant circadian variation was observed in young women but not in postmenopausal women. HSFA showed no significant circadian variation in either group. The results of their linear mixed‐effects model and circadian parameters are summarized in Tables A4 and A5.

## 4 DISCUSSION

The objective of this study was to investigate postmenopausal changes in sleep microstructure and its circadian variation. During specific thirds of the baseline sleep period, we found that postmenopausal women had a lower power within the delta and sigma ranges, and increased power within the alpha range. During the USW procedure, postmenopausal women had a dampened or undetectable circadian amplitude of delta (or SWA), theta and most frequencies in the sigma power (12–15 Hz). Additionally, we found that postmenopausal women had lower levels of delta, theta and bins within the sigma power (14–15 Hz). The nocturnal increase in sigma band power was also lower relative to daytime naps in postmenopausal women.

### 4.1 Sigma power

During the USW procedure, main findings in sigma power of postmenopausal compared to young women include the absence of rhythm in the 12–13 Hz and 14‐15 Hz bin, a dampened amplitude of the 13–14 Hz bin, and a decreased power in the 14–15 Hz bin. In prior studies, SFA has been classified into LSFA and HSFA, which follow different circadian rhythms: LSFA peaking at night and HSFA peaking during the day (Dijk et al., 1997; Munch et al., 2010).

In the current study, we observed a clear circadian variation of LSFA with a nocturnal peak in young women (Figure A1), whereas no rhythm was observed in postmenopausal women. This is consistent with other reports with similar LSFA definitions (Munch et al., 2010: 12.25–13.75 Hz; Dijk et al., 1997: 12.25–13 Hz). In a forced desynchrony protocol of seven young men, Dijk et al. (1997) found a circadian variation of LSFA peaking along with melatonin secretion. Munch et al. (2010) studied 24 men and women aged 55–78 years in a forced desynchrony protocol, and did not observe a significant circadian modulation of LSFA in their aged population. Although somewhat consistent with our LSFA results in postmenopausal women, their study included both older men and women and did not consider menopausal status, thus limiting comparison. To clarify the circadian variation of SFA, we divided its frequencies into 1‐Hz bins. As expected, we observed that young women had significant rhythms in both the 12–13 and 13–14 Hz ranges, with peak power density at night. Interestingly, postmenopausal women showed  an absent circadian rhythm in the 12–13 Hz bands, but there was a significant rhythm at 13–14 Hz of decreased amplitude and peak power density at night. It has been previously described that frequency within sleep spindles increases with aging (Purcell et al., 2017), which may explain the significant rhythm of 13–14 Hz in postmenopausal women, and the absence of one at 12–13 Hz. These group differences in LSFA observations are also obvious when nocturnal EEG power was plotted relative to its daytime value (Figure 2), where the peak of LSFA in postmenopausal women occurs at a higher frequency than in younger women (Dijk et al., 1997; Munch et al., 2005; Munch et al., 2010).

The absence of HSFA circadian variation in our study contrasts with previous findings. Dijk et al. and Munch et al. reported a circadian rhythm of HSFA in young men (13.75–15.5 Hz) and older men and women (14–15.5 Hz; Dijk et al., 1997; Munch et al., 2010), with the peak occurring during the day. While 14–15 Hz showed no circadian modulation in either group, 15–16 Hz presented a significant circadian variation in both postmenopausal and young women with a daytime peak. This 15–16 Hz variation, similar to the HSFA variation described in the literature, appeared at higher frequencies than prior studies (Dijk et al., 1997: men aged 21–25 years, 13.75–15.5 Hz; Munch et al., 2010: men and women aged 55–78 years, 14–15.5 Hz) and may indicate age and sex differences in this frequency band. However, more studies are needed to confirm this observation.

The absent and dampened circadian variation of sigma power (12–15 Hz) in postmenopausal women is consistent with an impaired output of the circadian pacemaker regulating spindle activity during sleep (Dijk & Duffy, 2020). Sleep spindles are part of sigma power and are considered a hallmark of NREM sleep, viewed as a protective factor to external stimuli and memory consolidation contributor (Fernandez & Luthi, 2020). They are generated in the reticular nucleus of the thalamus and integrated in the thalamocortical loop to reach the cortex (Fernandez & Luthi, 2020). Although it remains unknown whether the suprachiasmatic nucleus of the hypothalamus directly regulates the thalamocortical network, neuronal projections from the suprachiasmatic nucleus to the thalamus have been previously described (Novak et al., 2000) and the presence of a circadian variation of SFA further supports this connection.

Different processes may contribute to the lower levels and dampened circadian variation of SFA in postmenopausal women in the current study. Brain atrophy due to aging has been correlated with reduced sigma power (Guazzelli et al., 1986). Animal studies and clinical trials have observed enhanced sleep spindles with progesterone administration, possibly by stimulating the reticular nucleus of the thalamus (Belelli & Lambert, 2005; Fernandez & Luthi, 2020). We previously reported reduced melatonin in postmenopausal women (Perez‐Medina‐Carballo et al., 2023). Melatonin administration has also been shown to increase LSFA compared with placebo during daytime naps (Dijk et al., 1995). Consequently, brain atrophy, the declining levels of progesterone and melatonin, may contribute to the decreases in SFA and dampened rhythms of postmenopausal women, but the specific effect and contribution of these factors remain to be elucidated.

### 4.2 Delta power

Another interesting observation was the lower levels of SWA during the USW procedure and its blunted circadian variation in postmenopausal women compared with younger women. Although there were no differences between groups across the full delta frequency band (0.5–4.5 Hz), a lower power within the delta range was observed for postmenopausal women from 0.75 to 1.25 Hz during the first third of the baseline night, as well as the nighttime naps of the USW procedure. Given that SWA is understood to reflect the homeostatic sleep process (Borbely et al., 2016), the lower levels observed for postmenopausal women indicate a reduced accumulation of sleep pressure during time awake. To mitigate the confounding effect of sleep deprivation on SWA levels, both groups in this study followed a regular sleep–wake schedule with a similar duration of time in bed for 2 weeks prior to laboratory entry. In fact, they presented comparable TST, time in NREM sleep, and WASO during the baseline sleep period, and thus sleep deprivation prior to laboratory entry likely does not explain the group differences in SWA during the baseline sleep.

Although SWA is mainly controlled by the homeostatic process regulating sleep propensity, circadian modulation of SWA has also been demonstrated in both young (Dijk et al., 1997; Lazar et al., 2015; Santhi et al., 2016) and older (Munch et al., 2010) men and women. While the circadian variation of SWA seems to be reduced in older populations, we are not aware of prior studies that performed statistical comparisons between older and younger women. In addition to reduced production of SWA, consistent with a reduced sleep homeostatic process, the dampened rhythm of SWA in postmenopausal women during the USW suggests a weakened output of the circadian pacemaker.

The SWA is generated in the basal forebrain and distributed throughout the cortex and, accordingly, brain atrophy has been associated with decreased SWA with aging (Jones, 2020; Liu et al., 2017; Niethard et al., 2018). Estrogen administration has also been shown to decrease SWA in animal models and in one randomized clinical trial in humans (Kalleinen, Polo, et al., 2008; Smith et al., 2022). In the present study, young women had higher levels of SWA despite their higher levels of estrogen compared with postmenopausal women (Perez‐Medina‐Carballo et al., 2023). Although not directly tested in the current study, it is more likely that brain atrophy rather than lower ovarian hormone levels is the main contributor to the declined levels of SWA in this group of postmenopausal women based on the interpretation of the literature.

### 4.3 Theta power

Theta power also presented a significant circadian modulation in young women only. As a low‐frequency EEG wave, average levels of theta were also lower during the USW procedure in postmenopausal women. Although the literature on the circadian variation of theta power in NREM sleep is limited, our results are consistent with those showing a circadian variation in young (Dijk et al., 1997) but not in aged populations (Munch et al., 2010). Theta power is mainly modulated by the homeostatic process and affected by sleep deprivation. Therefore, as in the present study, it is expected to progressively increase in both groups during the USW procedure due to the slight built‐up sleep restriction (Cajochen et al., 1999). The function of theta power during NREM sleep remains unclear; however, during wakefulness and REM sleep, theta power has been associated with learning and memory consolidation (Karakas, 2020).

### 4.4 Alpha and beta power

Power in the high‐frequency bands, such as alpha and beta, has been associated with cortical arousal, sleep disruption and non‐restorative sleep (Ehrhart et al., 2000; Feige et al., 2013; Stone et al., 2008). Research on menopausal women found increased beta power linked to disrupted sleep, while alpha power has been less explored (Campbell et al., 2011; Matthews et al., 2021; Schwarz et al., 2017). In the current study, we found increased power within the alpha frequency band in postmenopausal women during the first third of the baseline sleep period. Likewise, during nighttime naps of the USW, increased power in frequencies of the beta range was observed at 24–24.5 Hz in postmenopausal compared with young women. Although the function of alpha power during NREM sleep remains unclear, it is possible that the increased alpha in postmenopausal women during the first part of the baseline sleep period relate to the increased number of arousals as reported in Perez‐Medina‐Carballo et al. (2023). In the current study, the increase in beta power during nighttime naps may reflect a relative state of hyperarousal at night in the postmenopausal group (Shi et al., 2022; Zhao et al., 2021), even though these women reported to be healthy sleepers. Interestingly, EEG power was comparable between groups during daytime naps, suggesting a possible influence of the endogenous circadian system.

In terms of circadian phase, we only observed differences between groups in the alpha rhythm, with postmenopausal women having an acrophase advanced by ~5 hr compared with young women. The significance of this finding remains to be elucidated. Although this advanced alpha rhythm may contribute to reinforce earlier sleep times in older women, the sleep schedule was only advanced by ~1 hr in postmenopausal compared with younger women. It also remains intriguing that no other EEG frequency bands showed phase differences. In a previous analysis of the current study, we did not observe phase differences in either melatonin, core body temperature and alertness rhythms between postmenopausal and young women (Perez‐Medina‐Carballo et al., 2023).

### 4.5 Strengths and limitations

Our study has limitations, including the small sample size, which increases the possibility of type 2 errors and limits the clinical implications of the study results. As such, during baseline sleep we were not able to observe group differences between standard frequency bands and group differences were only evident in the frequency bin analysis (by 0.25 Hz). Nevertheless, our results are consistent with an increased sleep fragility in postmenopausal women. We also included a postmenopausal woman taking hormone replacement therapy. However, because her results were within 2 SD of the group data, her data were deemed adequate to be included in the analyses. Additionally, women recruited in this study represent only a healthy minority of postmenopausal women, which may affect the generalizability of our results to the broader population at this stage of life.

Our results on SWA present two main limitations. First, only central channels (C3) were analysed, as frontal channels were not recorded in young women. The literature reports that the highest SWA activity is recorded in the frontal EEG channels (Lazar et al., 2015), which might influence our ability to detect between group differences in the present study. Second, we cannot completely exclude that the maintenance of an 8‐hr sleep schedule by participants prior to entry into the laboratory may have induced some sleep restriction. However, the groups were comparable at baseline in terms of several sleep parameters (TST, SE, and stages N2, N3 and REM sleep; Perez‐Medina‐Carballo et al., 2023), which suggests this risk is minimal. Moreover, the maintenance of a regular sleep schedule during the preparatory phase minimized the risk that the group differences observed in the circadian variation of sleep could be explained by differences in the regularity of their sleep–wake cycle.

Despite these limitations, our rigorous screening procedures allowed us to exclude several conditions that may affect the sleep and circadian rhythms of postmenopausal women, such as depression, sleep apnea, chronic conditions, use of medications, etc. Indeed, all our participants were free of medication apart from one postmenopausal woman taking hormone replacement therapy. Another strength of our study is the specialized protocol that allowed us to record sleep throughout the day and night for 48 hr, while minimizing masking factors that may affect our dependent variables. To the best of our knowledge, this is the first study to examine changes in the circadian variation of EEG power in postmenopausal women, adding important insights into the understanding of menopause‐related sleep changes.

## 5 CONCLUSION

In this small group of healthy‐sleeping postmenopausal women, we observed changes in the microstructure of sleep and its circadian variation. SFA differences may reflect aging processes such as hormonal changes at menopause, brain atrophy and declining melatonin levels, whereas SWA differences may largely reflect the effect of brain atrophy rather than that of ovarian hormones. Increased power within beta and alpha bands may reflect increased sleep disruption in this group of postmenopausal women. Furthermore, the dampened circadian variation of delta, theta and sigma power along with previous findings (Perez‐Medina‐Carballo et al., 2023) support the hypothesized weakened circadian signal promoting sleep in older women. Although our group of postmenopausal women were healthy and reported no sleep complaints, changes in their sleep microstructure underline the greater fragility of sleep at this vulnerable time of life, akin to that reported in patients with insomnia (Zhao et al., 2021). It is reasonable to hypothesize that the changes in EEG power frequency bands observed after menopause may contribute to the increased risk of developing sleep disturbances, although the observational design of our study does not allow us to assume causal relationships. Nevertheless, the present study clarifies the changes in EEG parameters that occur in women after menopause across circadian phases. These results may serve as a basis for the design of future epidemiological and clinical studies.

## AUTHOR CONTRIBUTIONS

**Rafael Pérez‐Medina‐Carballo:** Conceptualization; investigation; writing – original draft; methodology; validation; visualization; writing – review and editing; formal analysis; data curation; software. **Anastasi Kosmadopoulos:** Conceptualization; investigation; writing – review and editing; validation; methodology; formal analysis. **Christophe Moderie:** Writing – review and editing; visualization; formal analysis. **Philippe Boudreau:** Conceptualization; investigation; methodology; validation; visualization; writing – review and editing; software; formal analysis; data curation; resources. **Manon Robert:** Formal analysis; resources; visualization. **Diane B. Boivin:** Funding acquisition; conceptualization; investigation; supervision; project administration; methodology; validation; visualization; writing – review and editing; investigation.

## CONFLICT OF INTEREST STATEMENT

D.B.B. provides conferences and legal expert advice on sleep‐related topics.

## Supporting information


**DATA S1** Supporting information.

## Data Availability

The data underlying this article cannot be shared publicly because participants did not agree that their data be placed in a publicly accessible database. Therefore, for ethical and confidentiality reasons, the authors cannot provide public access to these data. Nevertheless, materials, data and protocols will be made available for investigation of scientific integrity if necessary. Readers are free to contact the principal investigator if they wish to discuss collaborations to build on these published data.
