# Supplementary material for: Dampened circadian amplitude of EEG power in women after menopause
Source: J Sleep Res. 2024 Apr 26;34(4):e14219. doi: 10.1111/jsr.14219 (PMC12215220; doi:10.1111/jsr.14219)
Supplement: Supplementary file 1 — DATA S1 Supporting information. [file JSR-34-e14219-s001.docx]

Supplementary tables and figures

**Dampened circadian amplitude of EEG power in women after menopause**

Rafael Pérez-Medina-Carballo^a,b^, Anastasi Kosmadopoulos^b,c^, Christophe Moderie^b^, Philippe Boudreau^b^, Manon Robert^b,d^, Diane B. Boivin^a,b^.

^a^ Integrated Program in Neuroscience, McGill University, Montreal, Quebec, Canada. H3A 1A1.

^b^ Centre for Study and Treatment of Circadian Rhythms, Douglas Mental Health University Institute, Department of Psychiatry, McGill University, Montreal, Quebec, Canada. H4H 1R3.

^c^ Appleton Institute for Behavioural Sciences, Central Queensland University, Adelaide, South Australia, Australia. 5034.

^d^ Centre de Recherche du Centre Hospitalier de l'Université de Montréal, Université de Montréal, Montreal, Quebec, Canada. H2X 0A9.

# **Appendix A**


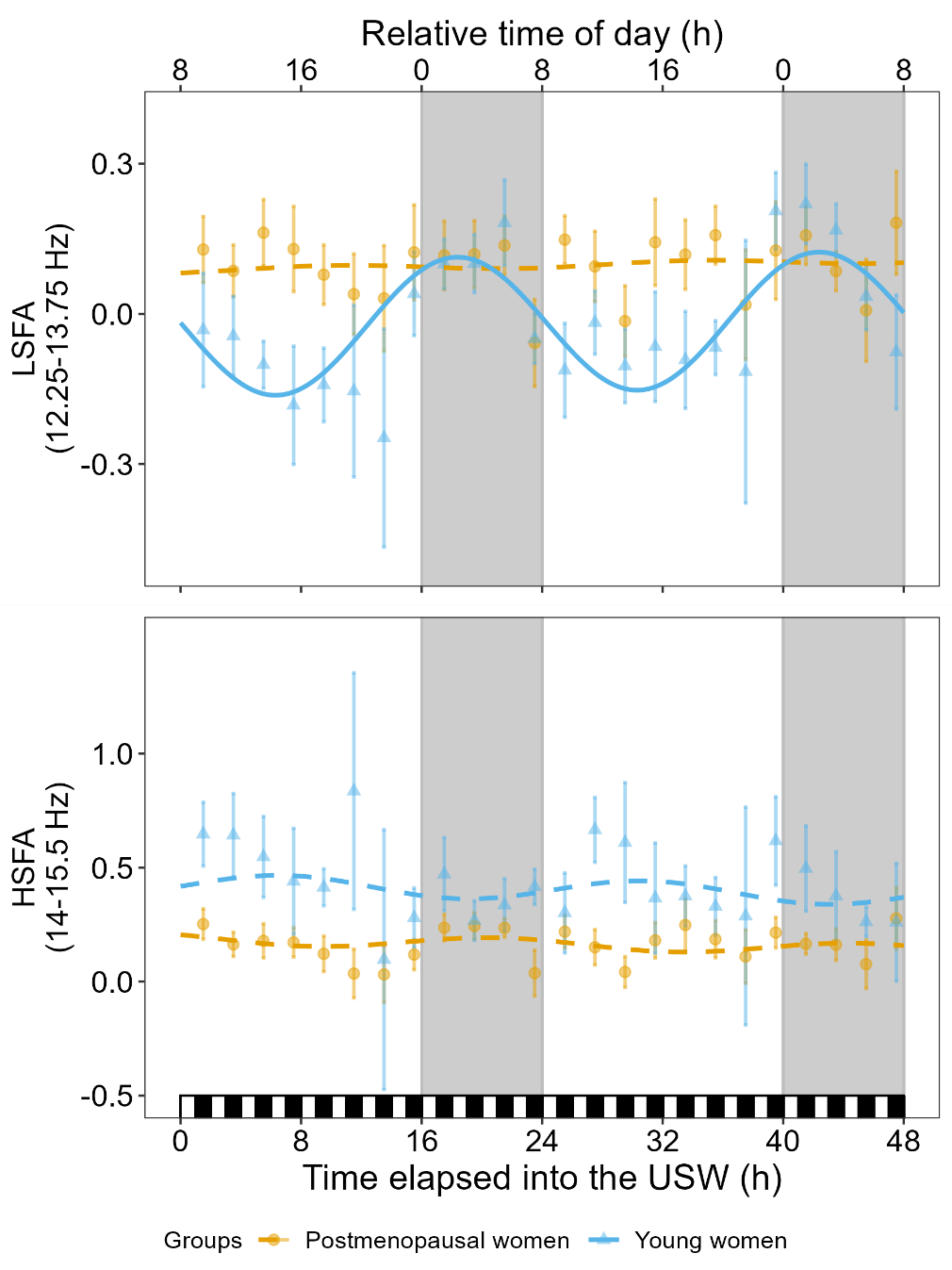


**Figure A1.** Circadian variation of low and high spindle frequency activity (LSFA, HSFA, respectively) throughout the USW procedure. Data were aligned on the time elapsed into the ultradian sleep-wake cycle procedure (USW, bottom X-axis). Solid lines represent significant cosinor regressions, whereas dashed lines depict non-significant regressions. Black (~0 lux) and white (~10 lux) small squares above the bottom X-axis represent the nap and wake periods across the first 48 hr of the USW procedure. The top X-axis depicts the corresponding time of day for a participant with a hypothetical bedtime of 00:00 hours to 08:00 hours. The Y-axis illustrates Z-scores. Large grey rectangles depict the projected time of the habitual nocturnal sleep period, corresponding to the time between habitual bedtime and rise time. Values are presented as mean ± SEM.

| **Participant** | **AHI** | **Participant** | **AHI** |
| --- | --- | --- | --- |
| YW-1 | 0 | PMW-1 | 2.05 |
| YW-2 | 0.68 | PMW-2 | 8.16 |
| YW-3 | 0 | PMW-3 | 6.02 |
| YW-4 | 1.44 | PMW-4 | 0.85 |
| YW-5 | 0 | PMW-5 | 2.96 |
| YW-6 | 0.4 | PMW-6 | 3.42 |
| YW-7 | 3.36 | PMW-7 | 5.36 |
| YW-8 | 0.95 | PMW-8 | 3.3 |
| YW-9 | 2.21 |  |  |
| YW-10 | 0 |  |  |
| YW-11 | N/A |  |  |
| YW-12 | 0 |  |  |
| **Mean** | **0.82** |  | **4.02** |
| **SD** | **1.11** |  | **2.36** |

**Table A1.** Apnea-hypopnea index (AHI) of postmenopausal (PMW) and young women (YW).

|  | **Mixed-model term** | | | | | |
| --- | --- | --- | --- | --- | --- | --- |
|  | Frequency band effect | | Group effect (postmenopausal vs young women) | | Group × Frequency band interaction | |
|  | X^2^ | p-value | X^2^ | p-value | X^2^ | p-value |
| Full SP | **6332.2** | **<0.001** | 0.38 | 0.54 | **173.03** | **<0.001** |
| 1^st^ third | **6357** | **<0.001** | 0.66 | 0.42 | **177.75** | **<0.001** |
| 2^nd^ third | **6152.6** | **<0.001** | 0.63 | 0.43 | **150.77** | **<0.001** |
| 3^rd^ third | **5694.2** | **<0.001** | 0.13 | 0.72 | **176.92** | **<0.001** |

**Table A2.** Summary of linear mixed-effects model for the full nocturnal baseline sleep period (SP) and divided by thirds. X^2^ and p-values are obtained from likelihood-ratio tests. Bold values denote statistical significance.

|  | Young women  (mean ± SEM) | Postmenopausal women  (mean ± SEM) | *p-value* |
| --- | --- | --- | --- |
| Delta power (µv^2^/0.25 Hz) |  |  |  |
| Full SP | 240.49 ± 28.48 | 189.15 ± 25.62 | 0.20 |
| 1^st^ third | 296.54 ± 25.54 | 232.90 ± 35.41 | 0.17 |
| 2^nd^ third | 140.12 ± 18.10 | 139.76 ± 25.688 | 0.84 |
| 3^rd^ third | 174.13 ± 76.29 | 92.87 ± 13.48 | 0.54 |
| Theta power (µv^2^/0.25 Hz) |  |  |  |
| Full SP | 13.98 ± 1.31 | 17.12 ± 1.61 | 0.15 |
| 1^st^ third | 15.74 ± 1.51 | 18.63 ± 1.88 | 0.25 |
| 2^nd^ third | 11.29 ± 1.41 | 14.79 ± 1.35 | 0.09 |
| 3^rd^ third | 11.47 ± 1.76 | 12.06 ± 1.24 | 0.49 |
| Alpha power (µv^2^/0.25 Hz) |  |  |  |
| Full SP | 10.09 ± 1.31 | 13.13 ± 2.49 | 0.30 |
| 1^st^ third | 10.02 ± 1.26 | 13.61 ± 2.83 | 0.27 |
| 2^nd^ third | 8.85 ± 1.35 | 11.21 ± 2.04 | 0.35 |
| 3^rd^ third | 8.48 ± 1.29 | 10.23 ± 2.03 | 0.48 |
| Sigma power (µv^2^/0.25 Hz) |  |  |  |
| Full SP | 7.98 ± 1.17 | 6.01 ± 0.84 | 0.19 |
| 1^st^ third | 7.12 ± 1.10 | 5.79 ± 0.80 | 0.34 |
| 2^nd^ third | 6.23 ± 0.88 | 5.20 ± 0.72 | 0.38 |
| 3^rd^ third | 6.56 ± 1.03 | 4.82 ± 0.66 | 0.18 |
| 12-13 Hz |  |  |  |
| Full SP | 2.04 ± 0.24 | 1.69 ± 0.28 | 0.35 |
| 1^st^ third | 2.02 ± 0.27 | 1.72 ± 0.30 | 0.46 |
| 2^nd^ third | 1.59 ± 0.17 | 1.44 ± 0.22 | 0.62 |
| 3^rd^ third | 1.50 ± 0.20 | 1.32 ± 0.22 | 0.55 |
| 13-14 Hz |  |  |  |
| Full SP | 2.78 ± 0.39 | 1.79 ± 0.34 | 0.07 |
| 1^st^ third | 2.46 ± 0.37 | 1.78 ± 0.33 | 0.19 |
| 2^nd^ third | 2.25 ± 0.33 | 1.55 ± 0.29 | 0.13 |
| 3^rd^ third | 2.06 ± 0.27 | 1.31 ± 0.21 | **0.042** |
| 14-15 Hz |  |  |  |
| Full SP | 2.24 ± 0.49 | 1.61 ± 0.19 | 0.25 |
| 1^st^ third | 1.85 ± 0.43 | 1.46 ± 0.16 | 0.09 |
| 2^nd^ third | 1.67 ± 0.36 | 1.41 ± 0.18 | 0.52 |
| 3^rd^ third | 2.01 ± 0.42 | 1.31 ± 0.18 | 0.15 |
| 15-16 Hz |  |  |  |
| Full SP | 0.92 ± 0.21 | 0.92 ± 0.13 | 0.66 |
| 1^st^ third | 0.79 ± 0.18 | 0.83 ± 0.12 | 0.44 |
| 2^nd^ third | 0.71 ± 0.14 | 0.80 ± 0.09 | 0.62 |
| 3^rd^ third | 1.00 ± 0.25 | 0.88 ± 0.13 | 0.66 |
| Beta power (µv^2^/0.25 Hz) |  |  |  |
| Full SP | 2.18 ± 0.34 | 2.35 ± 0.16 | 0.65 |
| 1^st^ third | 2.05 ± 0.30 | 2.42 ± 0.20 | 0.32 |
| 2^nd^ third | 2.24 ± 0.31 | 2.41 ± 0.16 | 0.64 |
| 3^rd^ third | 2.80 ± 0.57 | 2.64 ± 0.16 | 0.80 |

**Table A3.** Frequency bands for the full nocturnal baseline sleep period (SP) and divided by thirds. All values are expressed as mean ± SEM. *P*-values were based on two-tailed t-tests, or Mann-Whitney U tests when appropriate. Bold values denote statistical significance.

| Parameter | Group  (p-value) | Time into USW  (p-value) | Time into USW x group  (p-value) | Circadian variation  (p-value) | Circadian x Group Interaction  (p-value) |
| --- | --- | --- | --- | --- | --- |
| LSFA | 0.10 | 0.31 | 0.49 | **0.010** | **<0.001** |
| HSFA | 0.056 | 0.38 | 0.58 | 0.91 | 0.65 |

**Table A4.** Summary of linear mixed-effects model results of low and high spindle frequency activity (LSFA, HSFA, respectively) during the USW procedure. Bold values denote statistical significance.

| Parameter | Mesor (z-score) | | | | | Amplitude (z-score) | | | | | | | | Acrophase (elapsed time  into the USW in hr) | | | | |
| --- | --- | --- | --- | --- | --- | --- | --- | --- | --- | --- | --- | --- | --- | --- | --- | --- | --- | --- |
|  | PMW | | YW | | p-value | PMW | | | YW | | | p-value | PMW | | | YW | |  |
|  | Mean | SEM | Mean | SEM |  | Mean | SEM | 95% CI | Mean | SEM | 95% CI |  | Mean | | SEM | Mean | SEM | p-value |
| LSFA | 0.097 | 0.054 | -0.020 | 0.047 | 0.10 | 0.006 | 0.025 | -0.044, 0.055 | **0.135** | **0.022** | **0.092, 0.179** | **<0.001** | - | | - | 18.340 | 0.682 | - |
| HSFA | 0.164 | 0.095 | 0.403 | 0.082 | 0.06 | - | - | - | - | - | - | - | - | | - | - | - | - |

**Table A5.** Circadian parameters of low and high spindle frequency activity (LSFA, HSFA, respectively) throughout the USW procedure. Circadian parameters were calculated on Z-scores relative to their value during the baseline sleep period. Negative values represent negative changes relative to the baseline sleep period. Amplitude and phase were not calculated when the circadian variation was not significant. The *P-value* for mesor is equivalent to the group effect in *Table A4*. P-values were based on a two-tailed t-test. Bold values denote statistical significance. PMW = postmenopausal women; YW = young women.
